# Potential use of the S-protein–Angiotensin converting enzyme 2 binding pathway in the treatment of coronavirus disease 2019

**DOI:** 10.3389/fpubh.2022.1050034

**Published:** 2022-11-28

**Authors:** Long Feng, Shihui Fu, Pei Zhang, Yujie Zhang, Yali Zhao, Yao Yao, Leiming Luo, Ping Ping

**Affiliations:** ^1^Department of Anesthesia, Hainan Hospital of Chinese People's Liberation Army General Hospital, Sanya, China; ^2^Department of Geriatric Cardiology, Chinese People's Liberation Army General Hospital, Beijing, China; ^3^Department of Cardiology, Hainan Hospital of Chinese People's Liberation Army General Hospital, Sanya, China; ^4^School of Life Science, Beijing Institute of Technology, Beijing, China; ^5^Department of Epidemiology, School of Public Health, Southern Medical University, Guangzhou, China; ^6^Central Laboratory, Hainan Hospital of Chinese People's Liberation Army General Hospital, Sanya, China; ^7^Center for Healthy Aging and Development Studies, National School of Development, Peking University, Beijing, China; ^8^General Station for Drug and Instrument Supervision and Control, Joint Logistic Support Force of Chinese People's Liberation Army, Beijing, China

**Keywords:** angiotensin converting enzyme 2, coronavirus disease 2019, receptor binding domain, S-protein, 3E8

## Abstract

Severe acute respiratory syndrome coronavirus 2 (SARS-CoV-2), the pathogen that causes coronavirus disease 2019 (COVID-19), infects humans through a strong interaction between the viral spike protein (S-protein) and angiotensin converting enzyme 2 (ACE2) receptors on the cell surface. The infection of host lung cells by SARS-CoV-2 leads to clinical symptoms in patients. However, ACE2 expression is not restricted to the lungs; altered receptors have been found in the nasal and oral mucosa, vessel, brain, pancreas, gastrointestinal tract, kidney, and heart. The future of COVID-19 is uncertain, however, new viral variants are likely to emerge. The SARS-CoV-2 Omicron variant has a total of 50 gene mutations compared with the original virus; 15 of which occur in the receptor binding domain (RBD). The RBD of the viral S-protein binds to the human ACE2 receptor for viral entry. Mutations of the ACE2–RBD interface enhance tight binding by increasing hydrogen bond interactions and expanding the accessible surface area. Extracorporeal membrane oxygenation, hyperbaric oxygen, and aggressive dialysis for the treatment of COVID-19 have shown various degrees of clinical success. The use of decoy receptors based on the ACE2 receptor as a broadly potent neutralizer of SARS-CoV-2 variants has potential as a therapeutic mechanism. Drugs such as 3E8 could block binding of the S1-subunit to ACE2 and restrict the infection of ACE2-expressing cells by a variety of coronaviruses. Here, we discuss the development of ACE2-targeted strategies for the treatment and prevention of COVID-19.

## Introduction

Severe acute respiratory syndrome coronavirus 2 (SARS-CoV-2)—the causative pathogen of coronavirus disease 2019 (COVID-19)—is an enveloped non-segmented positive strand ribonucleic acid (RNA) virus belonging to the family *Coronaviridae*, order *Nidovirales*. SARS-CoV-2 infects upper respiratory tract cells and lung epithelial cells and can be detected in lower respiratory tract samples (1–3). Upper and lower respiratory symptoms are seen in individuals with COVID-19, and transmission occurs before symptoms develop. Typical clinical features are fever, sore throat, dry cough, rhinorrhea, sneezing, dyspnoea, headache, pneumonia, and increased cytokine levels (4). Severely infected individuals may succumb to severe sepsis and acute respiratory distress syndrome. In 2020, a global COVID-19 outbreak gave rise to public health concerns and warnings by the World Health Organization (5).

**Figure 1:**
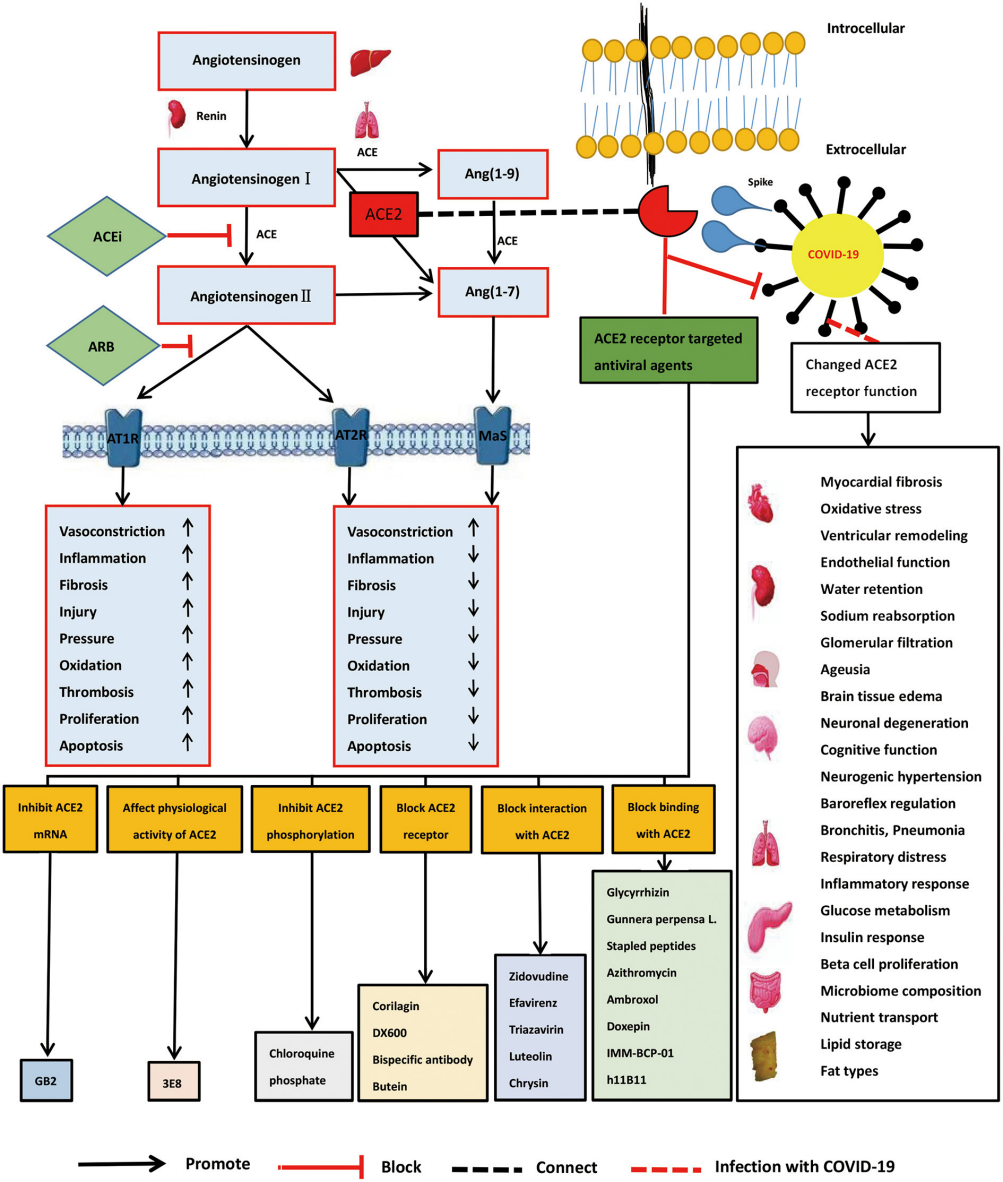
Role of angiotensin converting enzyme 2 (ACE2) receptor, ACE2 mechanism of viral action, and development of ACE2-targeted strategies in severe acute respiratory syndrome coronavirus 2 (SARS-CoV-2) infection.

Coronaviruses are prevalent and widely distributed, in part attributable to their genetic diversity and genome reassortment, and cross-species infection and occasional spillover (6). Coronaviruses have an error prone RNA-dependent RNA polymerase, resulting in frequent mutation and reassortment events. For example, mutation of the SARS-CoV-2 virus has resulted in improved binding to its cellular receptors and optimized replication in human cells (7). Our knowledge of coronaviruses remains limited, however, and serious public health threats are likely to occur in the future (8). The current outbreak of COVID-19, and possible outbreaks of other coronaviruses in the future, indicate that exploration of innovative therapeutic strategies and methods is warranted (9). As one SARS-CoV-2 primary receptor, recognizing the role of ACE2 in different pathways would be key to evaluating the impact of SARS-CoV-2/ACE2 binding on the physiology of the organs and helping us find better diagnostic tools and therapeutic approaches of SARS-CoV-2 (10). It is currently documented that its mechanism of action may be related to the imbalance of renin-angiotensin-aldosterone system (RAAS) and kallikrein system (KKS) (11–13). Here, we discuss the development of angiotensin converting enzyme 2 (ACE2)-targeted strategies for the treatment and prevention of COVID-19 (Figure 1).

## Spike proteins and angiotensin converting enzyme 2 receptors

SARS-CoV-2 gains entry into sensitive cells and achieves human-to-human transmission *via* strong interactions between viral spike proteins (S-protein) and angiotensin converting enzyme 2 (ACE2) receptors on the cell surface (13). Wan et al. (14) discovered that ACE2 is the receptor for COVID-19. The S-protein–ACE2 binding pathway plays a significant role in human transmission and the pathogenic process of COVID-19 (15). The S-protein comprises two components: S1 with a receptor binding domain (RBD), and S2 with a fusion peptide (9). Sequence variation replacing Arg426 with Asn426 in the SARS-CoV-2 S-protein resulted in the loss of hydrogen bond interactions and an increase in binding free energy. In the S-protein, substituted residues at positions 442, 472, 479, and 487 did not alter the three-dimensional structure of the RBD domain and maintained certain van der Waals and electrostatic properties on the interaction interface (16). ACE2 is key for the entry of SARS-CoV-2 into HeLa cells and is concentrated in a small subset of type II alveolar cells (AT2).

ACE2 variants are strongly associated with susceptibility to COVID-19 infection (17–20). Thirty two variants of ACE2 have now been identified by studying Asian, American, African, and European populations (21), including seven hotspot variants (lys26arg, ile468val, ala627val, asn638ser, ser692pro, asn720asp, and leu731ile/leu731phe). Genetic variation among different populations affects ACE2 function (20). For example, analysis of ACE2 expression profiles in normal lung cells, revealed that ACE2 expression is higher in Asian men than in white or African populations (18). This result suggests that Asian men may be more susceptible to viral infection. Interference of viral transmission and pathogenicity by regulating the S-protein–ACE2 binding pathway in ACE2-expressing AT2 may be an effective strategy to prevent and treat COVID-19.

## Omicron and ACE2 receptors

The emergence of the SARS-CoV-2 Omicron variant in Botswana and South Africa has influenced vaccine effectiveness and antibody capacity (22). Omicron has 50 gene mutations compared with the original strain, with 15 mutations in the RBD of the S-protein that binds to the ACE2 receptor. Thirty mutations of the Omicron S-protein are distributed over all domains of the trimeric protein, where the mutated residues are involved in intramolecular interactions, making it more stable. Seven mutations occur on the interaction interface between the RBD and the ACE2 receptor complex, including two ionic interactions, eight hydrogen bonds, and seven van der Waals interactions (23). Molecular features that have resulted in rapid diffusion of the Omicron variant include an increase in antibody evasion and the retention of strong interactions at the ACE2 interface (24). Unlike the Alpha, Beta, and Gamma variants, in Omicron the RBD binds to the human ACE2 receptor with a similar affinity to the prototypical RBD, likely owing to immune escape and compensation by multiple mutations that are transmissible (25). The structural basis for the binding of RBD-specific mutations to ACE2 receptors was revealed through the complex structures of Omicron RDB–ACE2 and Delta RDB–ACE2 (26). The Omicron RBD binds more strongly to the ACE2 protein, mainly through increased hydrogen bonding interactions and an enlarged accessible surface area (27).

## ACE2 location and symptoms

Although infection of host lung cells by SARS-CoV-2 can result in severe symptoms in patients, ACE2 expression is not unique to the lungs and altered receptors have been found in other tissues (28–30). ACE2 is highly expressed in the oral cavity, which increases the risk of COVID-19 infection (30). In addition, the expression of ACE2 decreases as the virus replicates, which causes prolonged constriction of the arteries, leading to increased dysfunction and inflammation, thereby resulting in significant cardiovascular damage (31). Symptoms such as “COVID toes” (chilblain-like acral lesions), reported during the COVID-19 pandemic, are likely a result of impairment of the vasculature (32). High expression levels of ACE2 in the heart and kidneys makes them susceptible to infection (33). Among hospitalized patients severely infected with this virus, 58% had hypertension and 44% had arrhythmias (8). ACE2 is highly expressed at the site of insulin production in the pancreas—decreased insulin secretion and altered glucose tolerance are associated with ACE2 deletion (34)—and diabetes has been identified as a unique comorbidity of COVID-19 (28). ACE2 is also highly expressed in the brain, especially in the glial cells, neurons, and spinal fluid (35). Brain tissue edema and partial neuronal degeneration have been found during autopsy of patients with COVID-19.

## ACE2 pathway and treatment

The renin–angiotensin II–aldosterone system plays a very important role in the body's regulation of circulatory and fluid homeostasis. Angiotensin II has immunomodulatory effects in the local pulmonary renin–angiotensin II system and the ACE/ACE2 balance is important for regulating angiotensin II levels. A homolog of ACE, ACE2 generates angiotensin 1–7 from angiotensin II. ACE2 plays an opposing role to ACE by counter balancing angiotensin II type 1 receptor (AT1R)-mediated actions and negatively regulates angiotensin II levels (10). Increased ACE and reduced ACE2 activities have been suggested to increase clinical susceptibility to acute and chronic pulmonary diseases. Loss of ACE2 expression increases vascular permeability, causing pulmonary edema and worsening lung function (36).

As a receptor for SARS-CoV-2, the downregulation of ACE2, and upregulation of ACE, may play causal roles in COVID-19 pathogenesis (37). ACE inhibitors have been confirmed to reduce ACE ability and increase ACE2 ability, and may prove beneficial in the treatment of COVID-19. Treatment of acute lung injury with active recombinant ACE2 protein can improve symptoms (37). ACE inhibitors can act as significant immunomodulators and decrease systemic cytokine levels (38). ACE inhibitors can also protect cardiopulmonary function and even improve the long-term prospects of patients with pulmonary disease (39). Current strategies for the treatment of diabetes and hypertension include ACE inhibitor drugs, angiotensin II receptor blockers, human recombinant ACE2, endogenous ACE2 activators, and ACE2 gene therapy (40). Soluble ACE2 (sACE2) can mediate viral entry into cells. Recombinant human ACE2 is an exogenous sACE2 that can complement endogenous ACE2, which may be an important option for the effective treatment of COVID-19 (41).

Vaccines are being used for active immunization against COVID-19 and drug repurposing and convalescent plasma may also be feasible treatment options (42). However, vaccinated persons have been infected with Omicron, and post-vaccination sera showed poor neutralization of the variant (43). The binding strength of the Omicron RBD to ACE2 is two-fold higher than that of prototype SARS-CoV-2. Spike mutations have promoted receptor binding to infect the respiratory system, and impaired antibody binding to evade the immune response (44). Novel treatments, including cocktail therapies, may be needed to treat Omicron infection.

## Treatment potential using ACE2

Molecular detection and close surveillance are essential to identify potential COVID-19 cases and deliver timely treatment (45). During the first contact of a clinician with a suspected case, different diagnoses may be made based on clinical symptoms and rapid pathogen detection. Urgent measures include adopting the most effective control strategies to avoid viral transmission in the community. For critically ill patients, varying degrees of clinical success have been achieved using extracorporeal membrane oxygenation, aggressive dialysis, and hyperbaric oxygen. Remdesivir, an RNA polymerase inhibitor, is the first Food and Drug Administration-approved treatment (46). Broad-spectrum antivirals, such as lopinavir, ritonavir, remdesivir, and interferon beta, are being evaluated for activity against COVID-19 (47, 48). Corticosteroids are commonly used to treat severely symptomatic patients by reducing inflammation-induced lung injury. However, administration of corticosteroids showed no effect on mortality and can delay viral clearance (49). Therefore, corticosteroids should not be routinely administered as a systemic treatment for COVID-19. Further study is urgently needed to evaluate whether different antiviral drugs and systematic corticosteroid treatment are applicable for patients infected with COVID-19 (8).

The theoretical and clinical significance of the S-protein–ACE2 binding pathway in viral transmission and pathogenicity highlight its potential as a target for COVID-19 treatments. ACE/ACE2-targeted therapeutic strategies are a cornerstone of cardiovascular therapeutics, and the same methods may be valid for the treatment of pulmonary disease, promoting the concept of synchronous treatment of the heart and lungs. Further studies are needed to investigate the use of therapeutic drugs based on the S-protein–ACE2 binding pathway.

Spike-binding ACE2 decoys may be an effective treatment for this viral infection as a result of their enhanced affinity and neutralizing efficacy (44). The use of decoy receptors based on ACE2 as a widely effective neutralizer of SARS-CoV-2 variants could have a variety of therapeutic mechanisms (Table 1). Chen et al. (50) found that 3E8, an antibody against human ACE2, could block binding of the S1-subunit to ACE2 without affecting the physiological activity of ACE2 or causing severe toxicities in hACE2 “knock-in” mice. In addition, 3E8 may be a potent “broad-spectrum” blocker of multiple SARS-CoV-2 variants, such as Delta, Omicron, Alpha, Beta, Kappa, and Gamma, which utilize ACE2 as the entry receptor (51). Studies have explored the interaction of the SARS-CoV-2 S-protein RBD with the ACE2 receptor in three variants (Omicron, Delta, and WT). Despite the multiple mutations of Omicron and its relatively high viral spread, the calculated binding affinities of phytochemical limonoids and glycyrrhizic acid support that traditional medicines can be used to formulate adjunctive therapies to combat this variant (52, 53). The above treatment strategies may be potential antiviral agents for Omicron-infected patients.

**Table 1:** Development of angiotensin converting enzyme 2 (ACE2) targeted strategies for the treatment and prevention of coronavirus disease 2019.

**Name**	**Function**	**References**
3E8	ACE2-targeting monoclonal antibody blocked the S1-subunits and pseudo-typed virus constructs from multiple coronaviruses, without markedly affecting the physiological activities of ACE2 or causing severe toxicity in ACE2 “knock-in” mice	Chen et al. (50)
h11B11	ACE2-blocking monoclonal antibody	Du et al. (62)
GB-2	Inhibiting ACE2 mRNA expression and ACE2 and TMPRSS2 protein expression in HepG2 and 293 T cells without cytotoxicity	Wu et al. (63)
Chloroquine phosphate	Inhibiting terminal phosphorylation of ACE2	Al-Bari (64)
DX600	DX600 is a potent ACE2 inhibitor specific for only human ACE2	Pedersen et al. (65)
Luteolin (3,4,5,7-tetrahydroxy flavone)	Luteolin can interact with Glu37, Lys353, Ala386, Met383, and Phe356 on the ACE2 receptor while the critical SAR-CoV-2-ACE2 interaction by hydrogen bonds is formed by three of them (Glu37, Lys353, and Met383)	Shahbazi et al. (66)
Chrysin (5,7-dihydroxy-2-phenyl-4H-Chromen-4-one)	Chrysin can interact with the ACE2 through Ala348 and Arg393 by hydrogen and hydrophobic bonds, respectively. This drug can interact with the ACE2 in a compact and stable mode	Shahbazi et al. (66)
Corilagin	Blocking the fusion of spike-RBD to ACE2 receptors	Yang et al. (67)
Glycyrrhizin	Binding to the ACE2 receptor	Chrzanowski et al. (68)
Azithromycin and ambroxol	Blocking the ACE2 receptor	Alkotaji (69)
Doxepin	Inhibiting viropexis of Spike pseudovirus by blocking ACE2	Ge et al. (70)
Zidovudine and efavirenz	Suppressing 2019-nCoV infection of ACE2-HEK293T cells by interacting with ACE2	Wang et al. (71)
Triazavirin	The interactions between TZV and given viral structures or the ACE-2 receptor might effectively block both the entry of the pathogen into a host cell and its replication	Hudecová (72)
Bispecific Antibody	Blocking the ACE2 receptor by linker cleavage inside the infected host	Ojha et al. (73)
Butein	Binding with ACE2 receptor	Kapoor et al. (74)
Stapled peptides	Inhibiting the binding of ACE2 receptor	Tzotzos (75)
IMM-BCP-01	Directly blocking Spike binding to the ACE2 receptor	Nikitin et al. (76)
Gunnera perpensa L.	Inhibiting SARS-CoV-2 spike glycoprotein-host ACE2 binding	Invernizzi et al. (77)

Omicron is characterized by high transmissibility and rapid spread, but its symptoms are less severe than those of previous variants. Early and prudent preventive measures, including vaccination, are key to inhibiting the Omicron variant (54). A recent study found that three doses of messenger RNA vaccine were more effective against the Omicron and Delta variants than not vaccinating or receiving two doses (55). Mutations in the S gene may generate novel variants with improved viral fitness through selective or survival advantages, such as increased ACE2 receptor affinity, replication, transmissibility, infectivity, immune escape, resistance to neutralizing antibodies, or disease severity (56). Quantitative analysis of the genetic transformation rate of the virus showed that the modified candidate drug catechin gallate can be repelled by ACE2, indicating that further modification of medical candidate drugs could produce effective docking inhibitors (57). Therefore, potential new solutions based on the ACE2 pathway could involve bait receptors based on ACE2, or mutations of the S gene.

## Conclusion

To date, there have been more than 620 million confirmed cases of COVID-19 worldwide and more than 6 million people have died (58). Since the outbreak of the pandemic, vaccines have been developed and administered, and the disease has been controlled to some extent. However, as the future of COVID-19 is uncertain, new viral variants may continue to emerge (59). Omicron showed 30 amino acid mutations in the S-protein, escaped the immunity of vaccinated individuals, and has shown increased infectivity and reinfection risk (Table 2) (60). Omicron has a lesser impact on the lower respiratory tract than previous variants and a reduced likelihood of hospitalization (61). Omicron remains infectious, however, and continues to influence work health policies and public health recommendations (54). New variants are likely to present new challenges for global control of COVID-19. Finding effective therapeutic drugs for COVID-19 is an urgent issue. In this context, therapeutic strategies that focus on the S-protein–ACE2 binding pathway are promising for treatment of COVID-19.

**Table 2:** Omicron showed 30 amino acid mutations in S-protein.

**Position**	**The spike protein of the virus**
In the N-terminal domain of the spike	N211del/L212I, Y145del, Y144del, Y143del, G142D, T95I, V70del, H69del, A67V
In the receptor-binding domain of the spike	Y505H, N501Y, Q498R, G496S, Q493R, E484A, T478K, S477N, G446S, N440K, K417N, S375F, S373P, S371L, G339D
In the fusion peptide of the spike	D796Y
In the heptad repeat 1 of the spike as well as multiple other mutations in the non-structural proteins and spike protein	L981F, N969K, Q954H

## Author contributions

All authors contributed to study design, data collection and analyses, and drafted whole paper.

## Funding

This work was supported by grants from the Military Medical Science and Technology Youth Incubation Program (20QNPY110 and 19QNP060), National Natural Science Foundation of China (81900357, 81941021, 81903392, 81901252, 82001476, 81802804, and 81801251), Excellent Youth Incubation Program of Chinese People's Liberation Army General Hospital (2020-YQPY-007), Natural Science Foundation of Hainan Province (821QN389 and 821MS112), Military Medicine Youth Program of Chinese People's Liberation Army General Hospital (QNF19069 and QNF19068), National Key R&D Program of China (2018YFC2000400), National S&T Resource Sharing Service Platform Project of China (YCZYPT[2018]07), Specific Research Fund of The Innovation Platform for Academicians of Hainan Province (YSPTZX202216), Hainan Major Scientific and Technological Cooperation Project (2016KJHZ0039), China Postdoctoral Science Foundation funded project (2019M650359, 2020M682816, and 2021T140298), Medical Big Data R&D Project of Chinese People's Liberation Army General Hospital (MBD2018030), National Geriatric Disease Clinical Medicine Research Center Project (NCRCG-PLAGH-2017-014), Central Health Care Scientific Research Project (W2017BJ12), Hainan Medical and Health Research Project (16A200057), Sanya Medical and Health Science and Technology Innovation Project (2016YW21, 2017YW22, 2018YW11, and 2018YW16), and Clinical Scientific Research Supporting Fund of Chinese People's Liberation Army General Hospital (2017FC-CXYY-3009). The sponsors had no role in the design, conduct, interpretation, review, approval, or control of this article.

## Conflict of interest

The authors declare that the research was conducted in the absence of any commercial or financial relationships that could be construed as a potential conflict of interest.

## Publisher's note

All claims expressed in this article are solely those of the authors and do not necessarily represent those of their affiliated organizations, or those of the publisher, the editors and the reviewers. Any product that may be evaluated in this article, or claim that may be made by its manufacturer, is not guaranteed or endorsed by the publisher.
